# The Re-organization of the Health Department

**Published:** 1887-01

**Authors:** 


### Editorial.

### *THE RE-ORGANIZATION OF THE HEALTH DEPARTMENT.*

By invitation of the present Board of Health, the members of the medical profession of this city were invited to meet at the rooms of the Board, and to give their views as to the re-organization of the Health Department. The sentiment expressed, both by the present Board and all present, was, that this city needed a more efficient organization. The manner in which this was to be accomplished was not so easily decided. Much useless and unprofitable discussion was indulged in, so that the people and the press got the impression that there was another doctors' row in progress. However spirited the discussion was, it must in justice be said that it was simply an honest and fair expression of opinion, free from any tendency to private quarrel. There were those who eloquently pleaded for a scientific Medical Board. Again, a plan was proposed for a body consisting of two laymen and three physicians. This seemed to be the plan in favor, until Dr. Briggs, by his convincing arguments, proved, to the satisfaction of many, that the City Engineer and the President of the Common

Council were greatly needed in such an organization. The Doctor evidently overlooked the fact that he is not always to be the Health Officer of the city—though we wish he might be; and that ordinarily such men have, by their indifference and political intrigues, been a hindrance in these organizations. The Doctor says he has found this to be quite to the contrary, and on this point he is to be congratulated. This has not been the good fortune of many medical officers of other cities. One physician of good standing, who has had considerable experience in health matters, favored a non-Medical Board, and still another would make the representation from the profession quite small. But we call to the mind the facts that such Boards have made many errors; in allowing nuisances to exist; in condemning as nuisances places that were not, etc., etc. From time to time, too, appointments to subordinate positions have been given to incompetent men. Such mistakes as these could not have been committed by medical men. Give us, then, a Medical Board. Do away with a body that condemns itself. Give us a Board composed of men that can advise and assist the Health Officer; that can supervise every department, and rich will be the reward. A Board composed entirely of medical men has many objections. First, it will not have the confidence of the people. Again, would the workings of such an organization be harmonious? Never! At least, never in this city. If a Mayor could choose five medical men, who would represent the varied interests of the profession in this city, and work in harmony, he would be the most phenomenal man ever elected to office. For these, and other reasons, we are greatly in favor of a mixed body, with a majority of medical men. To do effective committee work, the number should be five; committees of two, having charge of various departments. If such disposition of the work had been made in the past, the Committee in charge of the department of chemistry would have learned that their chemist was, for many weeks, in a distant city, and, practically, non-resident most of the time. The Committee having the supervision of the inspectors might have discovered that certain of the inspectors had not been *very* active in the performance of their duties. Who should appoint the members of such a Board? Were it within its power, they should be appointed by the Superior Court of Buffalo; such not being the case, we can rely, as a general thing, on the appointments of the Mayor as being of a high order.

It was proposed, in the outset, to make only those physicians eligible who have practiced ten years. This is certainly quite right. Only in this manner can men of experience be assured. This Board should appoint a Health Officer and various subordinates. It was the general sentiment of the profession that the Health Officer should devote his whole time to the performance of his duties in this department, to pay him a fair salary—say five thousand dollars a year. Much could be accomplished in this manner. If this were adopted, several of the inspectors would not be needed. The following was adopted as the sentiment of the members present:

That the Board be composed of a citizen taxpayer, not a physician, and not holding a public office, and two physicians, of at least five years' practice. The members of the Board shall be appointed by the Mayor, their term of office being six years, the term of each expiring every two years; the member whose term shall first expire to be chairman of the Board. The following officers shall be appointed by the Board: A health physician, for six years, at $5,000 per year, he to be the executive officer of the Board; a sanitary engineer, for six years, whose salary shall not exceed $1,500; a clerk of the Board, at $1,200; a clerk of vital statistics, at $1,000; a city chemist, at $1,500; a cattle inspector, at $1,200; one or more inspectors of food, at $900 each; a keeper of the quarantine hospital, at $600; six sanitary inspectors, at $600 each; ten district physicians, whose salaries shall be fixed by the Board, and which, in their aggregate, shall not exceed $9,000; and such other officers and subordinates as from time to time the Board shall require, subject to the approval of the Mayor.

The recommendations in most cases were made by close votes. This should be taken into consideration by the committee that has the matter in charge. Give us a Board of five; three medical and two laymen—the physicians to have practiced at least ten years. All the members shall be taxpayers in this city. Such a Board would answer the requirements. To pay the members a small amount is also just. The other provisons as recommended are good. The only one that is open to objection is the large number of health inspectors. Rather give us two assistant health officers, at a liberal salary. To have, as at present, efficient medical inspection in one district and little or none in another, is but little use. To stamp out pests and infectious diseases in one street, and allow it to run wild in another, is not fair nor consistent. That such is the case at present, no one knows better than our health authorities.

That all should be required to pass a civil service examination goes without saying; the best man for the place, is the motto of the JOURNAL every time. Before another year rolls around, may it be our lot to have such a department.

